# Supplementary material for: Slowdown of growth controls cellular differentiation
Source: Mol Syst Biol. 2016 May 23;12(5):871. doi: 10.15252/msb.20156691 (PMC5289222; doi:10.15252/msb.20156691)
Supplement: Supplementary file 1 — Appendix [file MSB-12-871-s001.pdf]

Appendix

Slowdown of growth controls cellular differentiation

Jatin Narula*[1], Anna Kuchina*[2], Fang Zhang[2], Masaya Fujita[3], Gürol M. Süel‡[2] and Oleg A. Igoshin‡[1]

[1]Department of Bioengineering, Rice University, [2]Division of Biological Sciences, UCSD,
[3]Department of Biology and Biochemistry, University of Houston

___

‡ authors to whom correspondence should be addressed

1. Appendix Figure 1

2. Appendix Tables A1-A3

3. Appendix Text A1: Mathematical Model of Sporulation Phosphorelay

4. Appendix Text A2: Population Dynamics Model of Growth and Sporulation

5. Appendix References

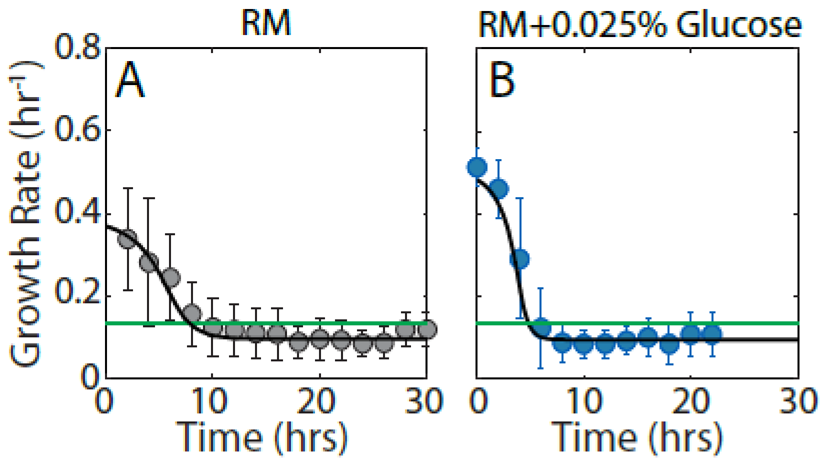

Appendix Figure 1. Effect of nutrient availability on growth slowdown and sporulation dynamics.

A. Dynamics of growth slowdown in normal starvation media (RM). Gray circles and errorbars show the mean and standard deviations respectively of the growth rate of a colony of cells in RM. In RM substrate availability is low from the start of the experiment. As a result, cells grow slowly and accordingly nutrient levels are depleted gradually until cells cross the growth threshold for sporulation (green line) around 10 hours into the experiment. Fitting this data (black curve) shows that these growth rate dynamics can be explained by a simple population dynamics model for substrate amount and number of cells (see Supplementary Text S2 for details).

B. Dynamics of growth slowdown with increased initial nutrients (RM+0.025% glucose). The population dynamics model predicts that growth rate dynamics are sensitive to initial nutrient availability. Glucose addition at the start of the experiment leads to the high initial growth rate and postpones starvation. However, subsequently, the increased number of cells results in rapid depletion of nutrients and decrease in growth rate (black curve). As a result, cells cross the growth threshold (green line) for sporulation earlier around 7 hours into the experiment. Experimental measurements of growth dynamics (blue circles) in RM+0.025% glucose confirmed the model predictions. Blue circles and errorbars show the mean and standard deviations respectively.

2. Appendix Tables

Table A1. *B. subtilis* strains used in this study

.

B. subtilis strains as referred in the article	*B. subtilis* strain number	Genotype	Used in Figures
"Wildtype", WT	AK151	*AmyE*::P$_{spo0A}$-*yfp*, P$_{comG}$-*mCherry* (Sp^R)<br>*SacA*::P$_{spoIIR}$-*cfp* (Cm^R)	Fig. 2D; Fig. S2; Fig. S3
	TC669	*AmyE::P$_{hsp}$-yfp* (Sp^R)	Fig. 2A; Fig. S1D-F
	AK2161	*P$_{spoIIR}$-YFP, P$_{spo0A}$-CFP, pDG148-P$_{rpsD}$-mCherry*	Fig. 1C-F; Fig. 3; Fig. S1H
	AK456	*AmyE*::P$_{spo0F}$-*yfp* (Sp^R)	Fig. S1A-C, G
	MF929	*KinA*::P$_{kinA}$-*kinA-gfp* (Kan^R)	Fig. 5CDE; Fig. S6
	AK2261	*AmyE*:: P$_{hsp}$-*DnaN-YFP* (Sp^R)<br>pHP13- P$_{spo0A}$-*cfp*, P$_{comG}$-*mCherry* (Erm^R)	Fig. S1I
iTrans-0F	AK2092	*AmyE*::P$_{hsp}$-*Spo0F* (Sp^R)<br>*SacA*:: P$_{spoIIR}$-*yfp* (Cm^R)<br>*GltA*::P$_{spo0F}$-*Spo0F* (Pm^R)<br>pHP13- P$_{spo0A}$-*cfp* (Erm^R)<br>*Spo0F*:: *kan* (Kan^R)	Fig. 4D-I
Inducible KinA	MF2840	*KinA::P$_{hsp}$-kinA–gfp* (Cm^R) (Kan^R)	Fig. 5CDE; Fig. S6
Sda deletion	F47-1	*sda::cm* (Cm^R*), AmyE::P$_{spo0A}$-yfp, P$_{comG}$-mCherry (Spec)*	Fig. S7

Table A2. Parameter values used for gene regulatory interactions in the model of sporulation phosphorelay.

**Reaction**	**Rate**	**Values**
$\rightarrow$ KinA	$v_{kinA} = v^0_{kinA} + v^{max}_{kinA} \frac{[S]^m}{K^m_{kinA} + [S]^m}$	v^0_{kinA} = 0.9 μM hr^{-1}, v^{max}_{kinA} = 1.5 μM hr^{-1}<br>K_{kinA}=0.025 μM, m = 1
$\rightarrow$ 0F	$v_{0F} = v^0_{0F} + v^{max}_{0F} \frac{[S]^m}{K^m_{0F} + [S]^m}$	v^0_{0F} = 0.15 μM hr^{-1}, v^{max}_{0F} = 3 μM hr^{-1}<br>K_{0F}=0.15 μM, m = 2
$\rightarrow$ 0B	v_{0B}	v_{0B} = 0.3 μM hr^{-1}
$\rightarrow$ 0A	$v_{0A} = v^0_{0A} + v^{max}_{0A} \frac{[S]^m}{K^m_{0A} + [S]^m}$	v^0_{0A} = 1.5 μM hr^{-1}, v^{max}_{0A} = 6 μM hr^{-1}<br>K_{0A}=0.35 μM, m = 2
$\rightarrow$ Rap	v_{rap}	v_{rap} = 0.075 μM hr^{-1}
$\rightarrow$ 0E	v_{0E}	v_{0E} = 0.03 μM hr^{-1}

Table A3. Parameter values used for the population dynamics model of growth dynamics during starvation.

Parameter	Description	Value
v_m	Maximum growth Rate	1.13 hr^{-1}
K_m	Half-maximal substrate concentration	0.82
k_d	Maximum sporulation rate	0.1 hr^{-1}
K_S	Half-maximal substrate concentration for sporulation	0.5
m	Hill-exponent for sporulation rate	3.5
γ	Substrate yield	0.02 substrate amt./cell
N_0	Initial Cell Number	SM: 5, SM+0.025% Glucose: 5
S_0	Initial Substrate Concentration	SM: 0.44, SM+0.025% Glucose: 0.72

3. Appendix Text A1: Mathematical Modeling Methods

Derivation of the dependence of protein concentrations on growth rate (Eq. [1])

To derive the Eq. [1] from the main text describing the dependence of protein concentration (C), on growth rate (μ) we started with differential equations for protein molecule number (N) including production and degradation terms and for exponential growth of cell volume (V):

$$\frac{dN}{dt} = P - k_{deg}N$$
$$\frac{dV}{dt} = \mu V$$

In the first equation, we include protein production (rate P) and degradation (rate k_{deg}). The second equation describes the exponential increase in cell volume with growth rate μ. Using these equations and definition for concentration C=N/V, we can derive the rate of change of C:

$$\frac{dC}{dt} = \frac{d}{dt}\left(\frac{N}{V}\right) = \frac{1}{V}\frac{dN}{dt} - \frac{N}{V^2}\frac{dV}{dt} = \frac{1}{V}\left(P - k_{deg}N\right) - \frac{N}{V}\mu V = \frac{P}{V} - \left(\mu + k_{deg}\right)C$$

At steady state this equation result in the postulated dependence of C on growth rate (Eq. [1] in the main text)

Phosphorelay network model

To investigate the dependence of 0A activity on cell growth rates we extended a previous mathematical model of sporulation phosphorelay network (Narula, Kuchina et al. 2015). This model used ordinary-differential equations describing concentration of the phosphorelay proteins and their complexes as a function of time to provide a deterministic description of the phosphorelay network response.

Our model can be subdivided into the following two parts: (i) the post-translational interactions that describe the phosphorylation/dephosphorylation of phosphorelay species and (ii) the transcriptional feedback interactions that control the expression of the phosphorelay proteins.

Post-translationally, the activity of the sporulation master regulator is controlled by the sporulation phosphorelay through phosphorylation/dephosphorylation reactions (Fig. 1B). Specifically, phosphoryl groups are transferred from the major sporulation kinase KinA to Spo0A (0A) via the phosphotransferases Spo0B (0B) and Spo0F (0F) (Hoch

1993, Eswaramoorthy, Duan et al. 2010). Phosphorylated 0F (0F~P) and 0A (0A~P) are subject to negative regulation by phosphatases Rap and Spo0E (0E), respectively. All post-translational reactions were modeled exactly as in (Narula, Kuchina et al. 2015) with mass-action kinetics and the rate constants that were estimated from the *in vitro* measurements of phosphorelay kinetics (Grimshaw, Huang et al. 1998).

Transcriptionally, the production of the phosphorelay genes *kinA*, *0F*, and *0A* is regulated by 0A~P (Fig. 1B) both directly and indirectly (via σ^H), thereby forming multiple feedback loops (Weir, Predich et al. 1991, Fujita and Sadaie 1998). For modeling the expression of phosphorelay proteins, we again followed (Narula, Kuchina et al. 2015) and assumed that rates of transcription can be modeled with appropriate Hill-functions. To model the delay induced by indirect feedback we assumed that 0A~P levels control the expression an intermediate regulator *S* which in turn controls the transcription of *kinA*, *0F*, and *0A* (similar to (Levine, Fontes et al. 2012)). The regulation of *kinA*, *0F*, and *0A* transcription intermediate regulators was modeled with the generic Hill-function:

$$v_p = v^0 + v^{max} \frac{[0A\sim P]^m}{K^m + [0A\sim P]^m}$$

Here v^0 and v^{max} represent the basal and maximal rate of transcription, respectively. K and m represent the half-maximal binding constant and the Hill-exponent, respectively. For simplicity, the rate of expression of the intermediate regulator was assumed to be linearly dependent on v_P(0A~P). For *spo0B*, *spo0E* and *rap* we assumed constant rates of transcription. The specific rate expressions and parameter values used are described in Table A2.

For the simulations of the inducible KinA strains (Figures 3CD), the *kinA* expression rate V_{kinA} was independent of 0A~P and varied between 0 μMhr^{-1} and 5 μMhr^{-1}. For the simulations of the *iTrans-0F* strain (Figures 4BC), the expression rate for the origin-proximal P_{hsp}*-0F* was independent of 0A~P and fixed at 0.7μMhr^{-1}. The protein degradation rate was constant for all proteins and was fixed at 0.3 hr^{-1}.

Growth and gene copy number dependence of transcription rate

The rates of expression of all genes in the model were assumed to be proportional to the gene copy number and cell growth rate according to the following equation:

$$v = g^* v_p / F(\mu)$$

Where v is the actual rate of gene expression, v_p represents the expressions described for each gene in Table A2, g is the gene copy number and F(μ) is a proportionality factor that models the effect of changes in cell size depending on the growth rate μ. F(μ) is normalized such that F=1 for cells with doubling time of 1 hour (μ=log(2) hr^{-1}). We used the following phenomenological expression for F(μ):

$$F(\mu)=a*\exp(b*\mu)+c$$

The values for a, b and c were determined by fitting the data for change in cell length at division as a function of growth rate (Fig. EV1H). We found that a=0.690, b=0.689 and c=0.745.

Simulations

All simulations of the phosphorelay response (Figs. 2, 5 and EV5) were done using the *ode15s* solver of MATLAB and a decreasing series of cell-cycle growth rates (μ) to mimic the starvation response in the experiments (compare Figs. 1 and 2B).

The cell-cycle durations, T_{cyc} were fixed based on the growth rates:

$$T_{cyc}=\log(2)/\mu \text{ hrs}$$

The DNA replication period duration T_{rep}, was also assumed to be growth rate dependent and we used the following phenomenological expression for T_{rep}:

$$T_{rep}=0.78+0.15/\mu \text{ hrs}$$

The values of the coefficients in the above equation were determined by fitting the data for change in DNA replication periods as a function of growth rate (Fig. EV1I). To identify DNA replication windows in time-lapse experiments we expressed a fluorescent DnaN-YFP fusion protein from the IPTG inducible P_{hsp} promoter and used the same quantification procedure as that described in (Narula, Kuchina et al. 2015).

For simplicity, replication was assumed to start immediately after cell-division.

All origin proximal genes (*0F*, P_{hsp}-*0F* in *iTrans-0F* and P_{0A} reporters) were assumed to be replicated at the start of the DNA replication period and all terminus proximal genes (*kinA*, *0B*, *0A* , 0F in *iTrans-0F* and P_{hsp}-*kinA-gfp* in the inducible KinA strain) were assumed to be replicated at the end of the DNA replication time-window.

For the signal dependent KinA activity hypothesis (Fig. 5A and Fig. EV4BDF), the KinA autophosphorylation rate, k_p was assumed to depend on the growth rate:

$$k_p=1+12/((5.8*\mu)^4+1) \text{ hr}^{-1}$$

For the signal independent KinA activity hypothesis (Fig. 5B and Fig. EV4CEG), the KinA autophosphorylation rate, k_p was fixed at 12 hr^{-1} and assumed to be independent of growth rate.

Dose responses of 0A~P pulse amplitudes

Under both signal-dependent and signal-independent KinA activity hypotheses, the 0A~P pulse amplitude, growth rate and KinA concentration during each cell-cycle were calculated from these simulations to determine the 0A~P pulse amplitude vs growth rate (Fig. 2D and Fig. EV4DE) and 0A~P pulse amplitude vs KinA concentration (Fig. EV4FG) dose response relationships.

To calculate the growth rate and KinA thresholds the 0A~P threshold was fixed at 0.9 μM and the dose response relationships were used to find the corresponding growth rate and KinA level. In the inducible KinA strain, the KinA and growth thresholds were calculated at different *kinA* production rates (V_{kinA}) to determine the interdependence of KinA and growth thresholds under the signal dependent and signal independent KinA activity hypotheses (Fig. 5AB).

Sensitivity of 0A~P pulse amplitudes

To calculate the sensitivity of 0A~P pulse amplitudes to variations in the phosphorelay protein levels (Fig. 2C) we tested the effect of increasing the production rate of the proteins on the 0A~P pulse amplitude. For each phosphorelay protein p, the production rate v_p, was increased by Δ=10% and then the 0A~P pulse amplitude $[0A\sim P]_\Delta$, was calculated at the growth rate $\mu=0.15hr^{-1}$ (corresponds to the $[0A\sim P]_{WT}=0.9\mu M$ – the sporulation threshold in our wildtype simulations). The normalized sensitivity of 0A~P pulse amplitudes to each phosphorelay protein p was then calculated using the following equation:

$$S_p = \frac{[0A\sim P]_\Delta - [0A\sim P]_{WT}}{\Delta * [0A\sim P]_{WT}}$$

Matlab code for growth dependent phosphorelay model simulations

```
function PhosphorelayGrowthModel
clc;clear;format('compact');
close all;

%Set Phosphorelay Parameters
```

```
pars=setpars;

%Calculate Initial Conditions
xii=zeros(1,20);
par1=pars;
par1(20)=0.5;par1(36)=2*pars(36);
[~,y]=ode15s(@eqnsint,[0 2e3],xii,[],par1);xi=y(end,:);

tsers=[];t0=0;ts=[];y1=[];kgrowths1=[];tss=[];tdivs=[];

% Set Growth History
kgs=logspace(log10(0.05),log10(0.5),12);
kgrowths=[0.5*ones(1,3),fliplr(kgs)];nrep=numel(kgrowths);

funtser=@(t,t2,st) logical(mod(t,st)>=0).*logical(mod(t,st)<t2);

%Growth dependence of gene expression rate
fvk=@(x) (3.466*exp(-log(2)./1)+3.743)./(3.466*exp(-log(2)./x)+3.743);
vind=[1 2 3 4 21 24 27 30 31];

%Growth dependence of DNA replication duration
fRepDuration=@(x) (0.15./x+0.78);

for i=1:nrep
    par1=pars;
    CellCycDuration=log(2)/kgrowths(i);
    RepDuration=fRepDuration(kgrowths(i));
    par1(20)=kgrowths(i);
    par1(vind)=pars(vind)*fvk(kgrowths(i));

    ts1=linspace(0,CellCycDuration,round(CellCycDuration)*100)';
    tser=funtser(ts1,RepDuration,CellCycDuration);
    tss=[tss;t0+ts1];tsers=[tsers;tser];
    tdivs=[tdivs t0+CellCycDuration];

    par2=par1;
    par2(36)=2*pars(36);
    [t,y]=ode15s(@eqnsint,[t0,t0+RepDuration],xi,[],par2);xi=y(end,:);

ts=[ts;t];y1=[y1;y];kgrowths1=[kgrowths1;kgrowths(i)*ones(size(t))];

    if CellCycDuration>RepDuration
        par2=par1;
        par2([35 36])=2*pars([35 36]);

[t,y]=ode15s(@eqnsint,[t0+RepDuration,t0+CellCycDuration],xi,[],par2);
xi=y(end,:);

ts=[ts;t];y1=[y1;y];kgrowths1=[kgrowths1;kgrowths(i)*ones(size(t))];
    end
```

```
    t0=t(end);
end

Ap=y1(:,13);

figure(1)
subplot(211)
map=0.5;box on;
area(tss,map*tsers,'FaceColor',0.9*ones(1,3),'EdgeColor','none');hold
on;
plot(ts,kgrowths1,'b');ylim([0 map]);xlim([0 max(ts)]);
line([tdivs;tdivs],[zeros(1,numel(tdivs));map*ones(1,numel(tdivs))],'L
ineStyle',':','Color','k');
set(gca,'XTick',0:10:max(ts),'YTick',0:.1:max(kgrowths1));
xlabel('Time(hrs)');ylabel('Growth Rate (hr-1)');
subplot(212)
map=2.5;
area(tss,map*tsers,'FaceColor',0.9*ones(1,3),'EdgeColor','none');hold
on;
plot(ts,Ap,'r');xlim([0 max(ts)]);ylim([0 map]);
line([tdivs;tdivs],[zeros(1,numel(tdivs));map*ones(1,numel(tdivs))],'L
ineStyle',':','Color','k');
set(gca,'XTick',0:10:max(ts));box on;
xlabel('Time(hrs)');ylabel('[0A~P] (\muM)');

function pars=setpars
%Phosphorelay Parameters
kb=5e3;kb2=1*kb;
ks=12;ksd=1;
k1=500;k2=300;k3=.5e3;k4=200;k5=800;
k6=200;k7=800;
k8=100;k9=100;k10=100;k11=100;
kdpa=2;kpa=0.05;
kdeg0=0.3;kdil=0.1;

vb=0.3;vr=0.075;ve=0.03;
vk=0.9;fk=1.5;Kk=.025;nk=1;
vf=.15;f0F=3;K0F=.15;nf=2;
va=1.5;f0A=6;K0A=0.35;na=2;
ngk=1;ngf=1;

pars=[vk,vf,vb,va,ks,ksd,kb,kb2,k1,k2,k3,k4,k5,k6,k7,k8,k9,k10,k11,kdi
l...
      f0F,K0F,nf,f0A,K0A,na,fk,Kk,nk,vr,ve,kdpa,...
      kdeg0,kpa,ngk,ngf];

function dx=eqnsint(~,x,pars)

pars=num2cell(pars);
```

```
[vk,vf,vb,va,ks,ksd,kb,kb2,k1,k2,k3,k4,k5,k6,k7,k8,k9,k10,k11,kdil,...
f0F,K0F,nf,f0A,K0A,na,fk,Kk,nk,vr,ve,kdpa,...
kdeg0,kpa,ngk,ngf]=deal(pars{:});

kdeg=kdeg0+kdil;kb3=kb;

Kt=x(1);Ft=x(2);Bt=x(3);At=x(4);Rt=x(5);Et=x(6);
Kp=x(7);KpF=x(8);
Fp=x(9);KF=x(10);
Bp=x(11);FpB=x(12);
Ap=x(13);BpA=x(14);
FpR=x(15);ApE=x(16);
sKt=x(17);sFt=x(18);sBt=x(19);sAt=x(20);

K=max(Kt-Kp-KpF-KF);F=max(Ft-Fp-KpF-KF-FpB-FpR);
B=max(Bt-Bp-FpB-BpA);A=max(At-Ap-BpA-ApE);
R=max(Rt-FpR);E=max(Et-ApE);

vkp=vk+fk*Ap^nk/(Kk^nk+Ap^nk);
vfp=vf+f0F*Ap^nf/(K0F^nf+Ap^nf);
vap=va+f0A*Ap^na/(K0A^na+Ap^na);

dx(1)=ngk*sKt-kdeg*Kt;
dx(2)=ngf*sFt-kdeg*Ft;
dx(3)=ngk*sBt-kdeg*Bt;
dx(4)=ngk*sAt-kdeg*At;
dx(5)=vr-kdeg*Rt;
dx(6)=ve-kdeg*Et;

dx(7)=ks*K-ksd*Kp-kb*Kp*F+k1*KpF-kdeg*Kp;
dx(8)=kb*Kp*F-(k1+k2+kdeg)*KpF+kb3*K*Fp;
dx(9)=k2*KpF-kdeg*Fp-kb*Fp*B+k4*FpB-kb*Fp*R+k8*FpR-kdpa*Fp-kb3*K*Fp;
dx(10)=kb2*K*F-(k3+kdeg)*KF;
dx(11)=-kdeg*Bp-kb*F*Bp+k5*FpB-kb*A*Bp+k6*BpA;
dx(12)=kb*(Fp*B+Bp*F)-FpB*(kdeg+k4+k5);
dx(13)=-kdeg*Ap-kb*B*Ap+k7*BpA-kb*Ap*E+k10*ApE-kdpa*Ap+kpa*A;
dx(14)=kb*(Ap*B+Bp*A)-BpA*(kdeg+k6+k7);
dx(15)=kb*Fp*R-(k8+k9+kdeg)*FpR;
dx(16)=kb*Ap*E-(k10+k11+kdeg)*ApE;

ep=1.*kdeg;
dx(17)=ep*(vkp-sKt);
dx(18)=ep*(vfp-sFt);
dx(19)=ep*(vb-sBt);
dx(20)=ep*(vap-sAt);
dx=dx';
```

4. Appendix Text A2: Population Dynamics Model of Growth and Sporulation

To understand the effect of glucose addition on cell growth rate dynamics and thereby sporulation (Fig. 3C-F), we built a simple population dynamics model. We assumed that cell growth rate during starvation follows Monod kinetics (Kovarova-Kovar and Egli, 1998). Based on the observation of (Veening et al., 2008), we also assumed that cell death/sporulation releases nutrients that can be reused for cell growth. Our model is given by two equations for the number of cells (N) and amount of substrate (S):

$$\frac{dN}{dt} = \left[v_m \frac{S}{K_m + S} - \frac{k_d}{1 + (S/K_S)^m} \right] N$$

$$\frac{dS}{dt} = -\gamma \left[v_m \frac{S}{K_m + S} - \frac{k_d}{1 + (S/K_S)^m} \right] N$$

Here v_m and K_m are the maximum growth rate and the half-maximal substrate concentration for the Monod growth kinetics, respectively. To model the growth threshold-based sporulation decision, we assumed that sporulation rate is a non-linear function of the available substrate concentrations. k_d, K_s and m are the maximum sporulation/death rate, half-maximal concentration and the Hill-exponent, respectively. Parameter γ is the substrate yield. These model parameters along with the initial substrate concentration (arbitrary units: amt. substrate) were determined by fitting the model to data for cell growth from time-lapse experiments in Resuspension Media. The Levenberg-Marquardt algorithm of MATLAB *fsolve* function was used for fitting. The initial cell number was fixed to 5. The parameter values determined from fitting are shown in Table A3. Using these same values for parameters, the model was used to explain the effect of increased nutrient availability at the start of the experiment.

As shown in Appendix Fig. 1, this model shows that the dynamics of cell growth are sensitive to the initial substrate availability. In regular sporulation media, substrate availability is low from the start of the experiment. As a result, cells grow slowly and the nutrient levels are accordingly depleted gradually until the cells cross the growth threshold for sporulation around 10 hours into the experiment (Appendix Fig. 1A). In contrast, the addition of 0.025% glucose at the start of the experiment increases the initial substrate availability and postpones starvation (Appendix Fig. 1B). Under these conditions cells grow rapidly and multiply. The increased number of cells at the onset of starvation results in a rapid depletion of nutrients and a decrease in growth rate (Appendix Fig. 1B). Consequently, in these conditions the cells start to sporulate earlier: around 7 hours into the experiment (Fig. 3B).

Matlab code for Population Dynamics Model of Growth and Sporulation

```
function BacillusPopGrowthModel
clc;clear;format('compact');
close all;

yield=0.02;Vm=1.13;Km=0.82;m=1;
kd=0.1;Ks=0.5;md=3.5;
s0RMglu=0.72;s0RM=0.44;n0=5;
par0=[yield,Vm,Km,m,kd,Ks,md];

tspan=[0 30];

[t,y]=ode45(@eqns,tspan,[n0 s0RM],[],par0);
N=y(:,1);S=y(:,2);kgRM=Vm*S.^m./(Km^m+S.^m);

figure(1)
subplot(221)
plot(t,kgRM,'k');hold on;axis square;
xlim([0 30]);ylim([0 0.8]);
xlabel('Time(hrs)');ylabel('Growth rate (hr-1)');title('RM');

[t,y]=ode45(@eqns,tspan,[n0 s0RMglu],[],par0);
N=y(:,1);S=y(:,2);kgRMglu=Vm*S.^m./(Km^m+S.^m);

subplot(222)
plot(t,kgRMglu,'r');hold on;axis square;
xlim([0 30]);ylim([0 0.8]);
xlabel('Time(hrs)');ylabel('Growth rate (hr-1)');
title('RM+0.025%Glu');

function dydt=eqns(t,x,pars)
yield=pars(1);Vm=pars(2);Km=pars(3);m=pars(4);
kd=pars(5);Ks=pars(6);md=pars(7);
N=x(1);S=x(2);

dydt(1)=(Vm*S^m/(Km^m+S)-kd/((S/Ks)^md+1))*N;
dydt(2)=-yield*(Vm*S^m/(Km^m+S^m)-kd/((S/Ks)^md+1))*N;
dydt(2)=logical(S>0)*dydt(2);

dydt=dydt';
```

4. Appendix References

Eswaramoorthy, P., D. Duan, J. Dinh, A. Dravis, S. N. Devi and M. Fujita (2010). "The threshold level of the sensor histidine kinase KinA governs entry into sporulation in *Bacillus subtilis*." J Bacteriol **192**(15): 3870-3882.

Fujita, M. and Y. Sadaie (1998). "Feedback loops involving Spo0A and AbrB in in vitro transcription of the genes involved in the initiation of sporulation in *Bacillus subtilis*." J Biochem **124**(1): 98-104.
Grimshaw, C. E., S. Huang, C. G. Hanstein, M. A. Strauch, D. Burbulys, L. Wang, J. A. Hoch and J. M. Whiteley (1998). "Synergistic kinetic interactions between components of the phosphorelay controlling sporulation in Bacillus subtilis." Biochemistry **37**(5): 1365-1375.
Hoch, J. A. (1993). "Regulation of the phosphorelay and the initiation of sporulation in Bacillus subtilis." Annu Rev Microbiol **47**: 441-465.
Levine, J. H., M. E. Fontes, J. Dworkin and M. B. Elowitz (2012). "Pulsed feedback defers cellular differentiation." PLoS Biol **10**(1): e1001252.
Narula, J., A. Kuchina, D. Y. Lee, M. Fujita, G. M. Suel and O. A. Igoshin (2015). "Chromosomal Arrangement of Phosphorelay Genes Couples Sporulation and DNA Replication." Cell **162**(2): 328-337.
Weir, J., M. Predich, E. Dubnau, G. Nair and I. Smith (1991). "Regulation of spo0H, a gene coding for the Bacillus subtilis sigma H factor." J Bacteriol **173**(2): 521-529.
