# Supplementary material for: Slowdown of growth controls cellular differentiation
Source: Mol Syst Biol. 2016 May 23;12(5):871. doi: 10.15252/msb.20156691 (PMC5289222; doi:10.15252/msb.20156691)
Supplement: Supplementary file 2 — Expanded View Figures PDF [file MSB-12-871-s002.pdf]

Expanded View Figures

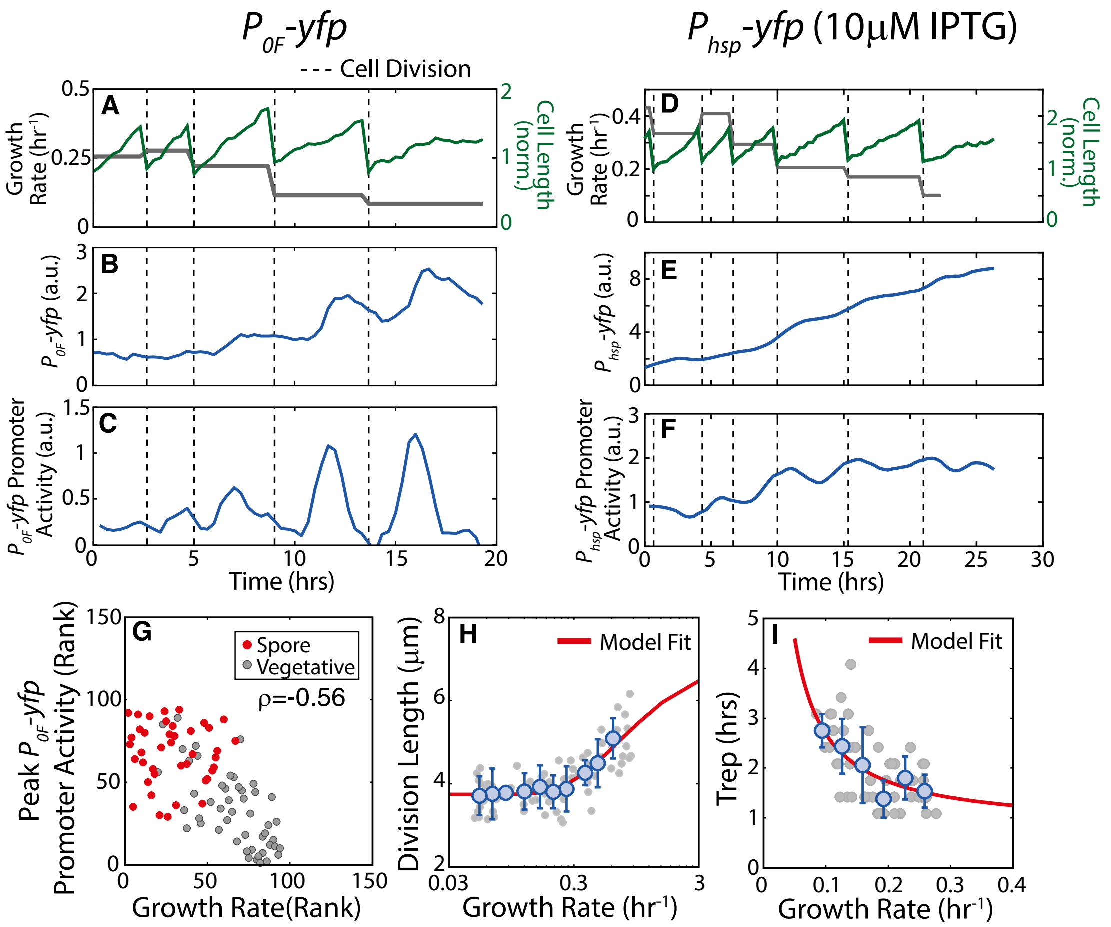

Figure EV1. Effect of growth slowdown on pulsing cell volume and DNA replication.

A Single-cell time-lapse microscopy measurements of cell length (green) and cell growth rate (gray), over multiple cell cycles in starvation media. In (A–C), vertical dashed lines indicate cell divisions.

B Fluorescence measurements for P_{OF}-*yfp* reporters in WT background show that the expression level of P_{OF}-*yfp* increases in non-monotonic fashion.

C Promoter activity of P_{OF}-*yfp* reporter shows pulses once every cell cycle. Promoter activity pulse amplitudes increase as growth rate decreases.

D–F Same as (A–C) except for an IPTG-inducible P_{hsp}-*yfp* reporter in WT background induced with 10 μM IPTG. Note that promoter activity of P_{hsp}-*yfp* reporter (F) does not shows pulses.

G Measurements of P_{OF}-*yfp* promoter activity show that the pulse amplitudes and growth rates are anti-correlated. Each dot corresponds to ranked measurements of the P_{OA}-*yfp* promoter activity pulse amplitude and growth rate of an individual cell cycle. Red and gray dots indicate cell cycle that ends in sporulation and vegetative division, respectively. The resulting Spearman's rank correlation $\rho = -0.56$, *P*-value $< 10^{-60}$, $N = 94$.

H Measurements of cell length at division show that cell size decreases with decreasing growth rate. Gray circles show cell lengths at division over 25 h of growth slowdown in starvation conditions ($N = 72$ individual cell cycles). Blue circles and error bars show the mean and standard deviations of division length measurements binned according to growth rate ($N > 3$ for each bin). Solid line indicates the phenomenological fit ($L(\mu) = 3.466*\exp(-0.689/\mu)+3.743$ μm) for cell length dependence on growth rate that we use in our models.

I Measurements of Trep, the duration of DNA replication period at different cell growth rates. Gray circles show Trep measured by detecting DnaN-YFP foci over 25 h of growth slowdown in starvation conditions ($N = 178$ individual cell cycles). Blue circles and error bars show the mean and standard deviations of Trep measurements binned according to growth rate ($N > 5$ for each bin). Solid line indicates the phenomenological fit ($Trep(\mu) = 0.15/\mu+0.78$ h) for DNA replication period dependence on growth rate that we use in our models.

Source data are available online for this figure.

 © 2016 The Authors

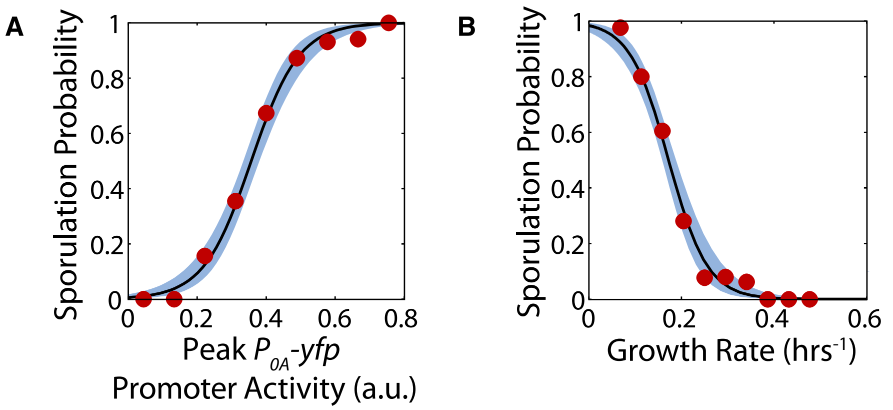

Figure EV2. Dependence of sporulation cell fate on P_{0A} promoter activity and growth rate.

A Probability of sporulation as a function of peak P_{0A}-*yfp* promoter activity calculated using logistic regression on the data in Fig 2D. Solid lines and blue areas indicate the logistic regression curves and the 95% confidence intervals. Red dots show the fraction of cells sporulating calculated by binning data in linearly spaced bins of P_{0A}-*yfp* promoter activity.

B Probability of sporulation as a function of growth rate calculated using logistic regression on the data in Fig 2D. Solid lines and blue areas indicate the logistic regression curves and the 95% confidence intervals. Red dots show the fraction of cells sporulating calculated by binning data in linearly spaced bins of growth rate.

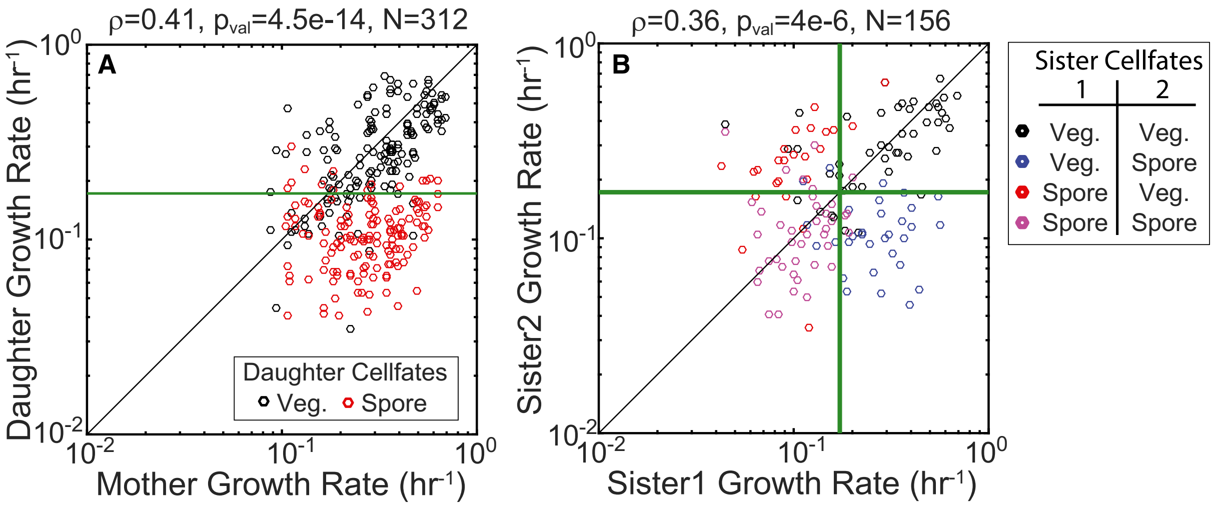

Figure EV3. Role of growth in determining sporulation cell fates for mother–daughter and sister cell pairs.

A Correlation plot showing growth rates of mother–daughter pairs during starvation. Each circle represents a single mother–daughter pair. Red and black circles indicate pairs for which the daughter cell sporulated or divided vegetatively, respectively. The green line shows the growth threshold below which daughter cells are expected to sporulate.

B Correlation plot showing growth rates of sister cell pairs during starvation. Each circle represents a single sister cell pair. Circles are colored according cell fates adopted by the sister pair. The green line shows the growth threshold below which cells are expected to sporulate.

Data information: Black lines in (A, B) represent $y = x$.
Source data are available online for this figure.

© 2016 The Authors

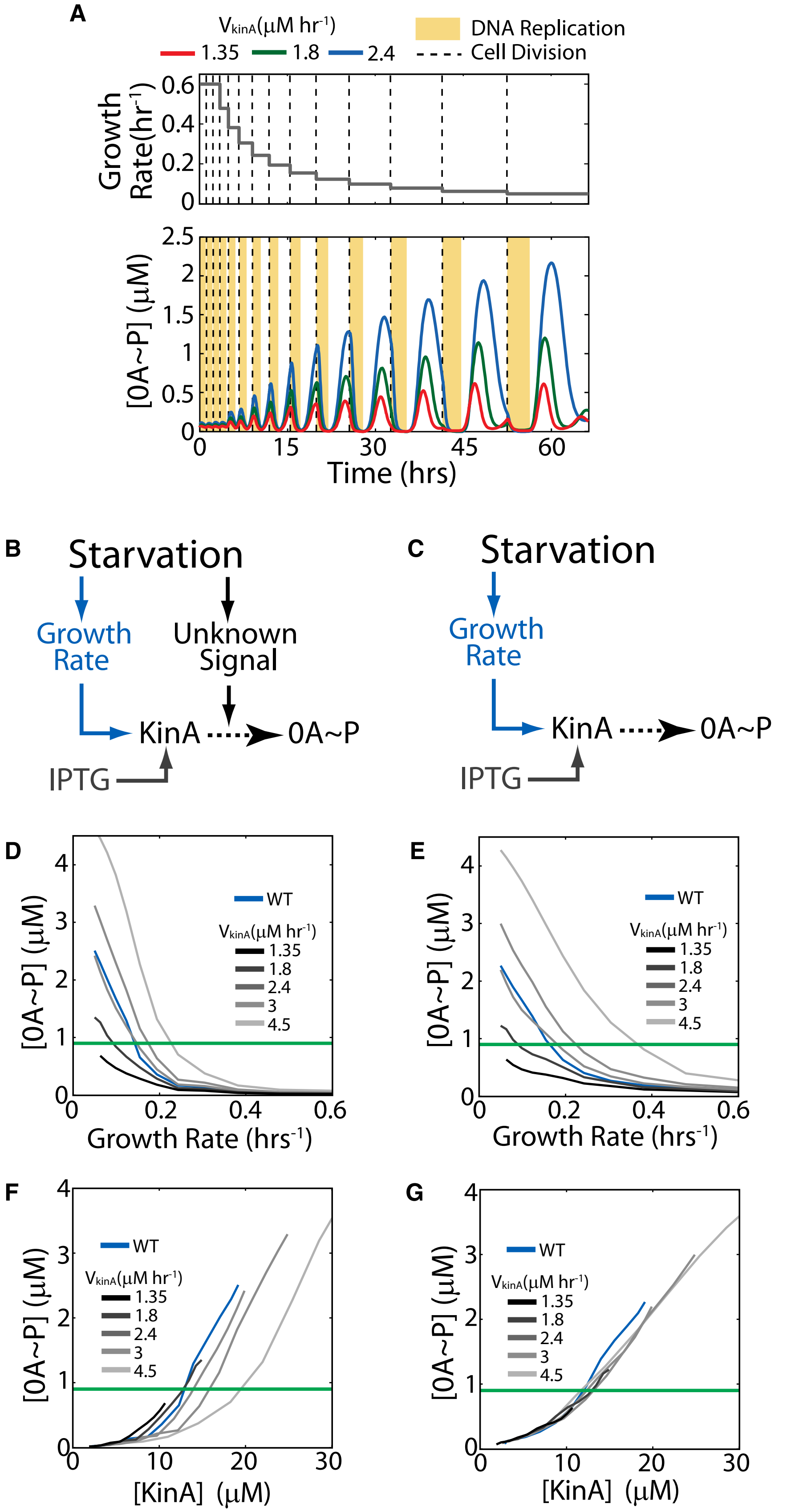

Figure EV4. Model predictions for 0A~P pulsing in an inducible KinA strain.

A Model time course for an inducible KinA strain showing cell-cycle-coordinated 0A~P pulsing. Upper and lower panels show the growth rate (model input) and 0A~P response (simulation result), respectively. Red, green, and blue curves show the 0A~P response at increasing rates of KinA production (V_{kinA}) from an inducible promoter. Yellow bars and dashed lines represent DNA replication periods and cell division, respectively. The simulation predicts that 0A~P pulses in the inducible KinA strain and that similar to WT, pulse amplitudes increase with decreasing cell growth rate and with increase in KinA induction.

B, C Two alternative hypotheses for the relationship between starvation, 0A~P, and sporulation cell fate. (B) Starvation controls 0A activation by affecting cell growth and by increasing KinA activity via an unknown signal. (C) Starvation controls 0A activation by affecting only cell growth but not KinA activity.

D, E Model predictions for the dependence of the 0A~P pulse amplitudes on growth rate under the signal-dependent (D) and signal-independent (E) KinA activity hypotheses. The green line shows the 0A~P threshold used in model simulations to predict cell fate. Note that the growth rate threshold (growth rate at which the 0A~P threshold is reached) depends on the KinA production rate (V_{kinA}) under both hypotheses.

F, G Model predictions for the dependence of the 0A~P pulse amplitudes on KinA under the signal-dependent (F) and signal-independent (G) hypotheses. The green line shows the 0A~P threshold used in model simulations to predict cell fate. Note that the KinA threshold (KinA level at which the 0A~P threshold is reached) depends on the KinA production rate (V_{kinA}) under the signal-dependent (F) but not the signal-independent (G) KinA activity.

© 2016 The Authors

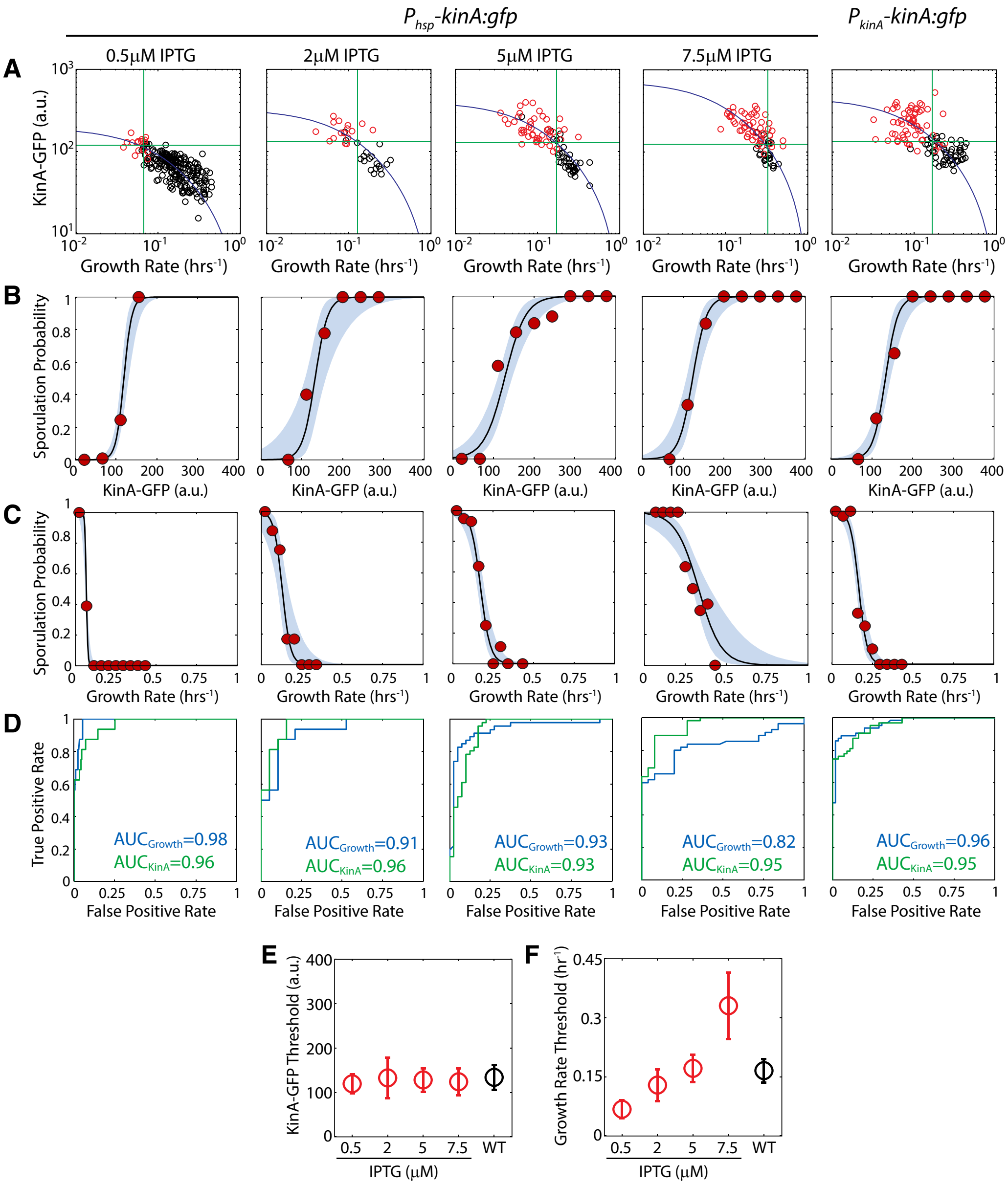

Figure EV5. Experimental measurement of the sporulation cell-fate outcomes as a function of KinA level and growth rate.

A KinA-GFP levels in the IPTG-inducible P_{hsp}-*kinA:gfp* (at different IPTG concentrations) and WT (P_{kinA}-*kinA:gfp*) as a function of cell-cycle growth rate. Measurements confirm that 0A activity KinA-GFP levels increase as growth rate decreases during starvation. Each dot corresponds to a single cell cycle. Black and red dots correspond to cell cycles that end in vegetative division and spores, respectively. Blue solid lines show the model predictions for KinA dependence on growth rate (same as that used in Fig 2A). Horizontal and vertical green lines show the thresholds that can be used to predict cell fate as a function of KinA level and growth rate, respectively.

B Probability of sporulation as a function of KinA-GFP levels calculated using logistic regression on the data in (A). Solid lines and blue areas indicate the logistic regression curves and the 95% confidence intervals. Red dots show the fraction of cells sporulating calculated by binning data in linearly spaced bins of KinA-GFP.

C Probability of sporulation as a function of growth rate calculated using logistic regression on the data in (A). Solid lines and blue areas indicate the logistic regression curves and the 95% confidence intervals. Red dots show the fraction of cells sporulating calculated by binning data in linearly spaced bins of growth rate.

D Receiver operating characteristic (ROC) curves for KinA-GFP-based and growth-based cell-fate prediction. Green and blue lines show the relation between false-positive rate and true-positive rate for different values of KinA-GFP and growth rate threshold.

E KinA-GFP thresholds calculated using the results of the logistic regression in (B). Error bars show standard errors. Significance of IPTG dependence of KinA threshold was determined by logistic regression of pooled data (N_{pooled} = 547) and applying a *t*-test to determine whether the regression coefficient for IPTG level is zero (see Materials and Methods for details). Note that the KinA threshold does not depend on IPTG level (*P*-value for IPTG coefficient = 0.70784, N_{pooled} = 547).

F Growth rate thresholds calculated using the results of the logistic regression in (C). Error bars show standard errors. Growth rate threshold increases with IPTG level (*P*-value for IPTG coefficient = 2.601e-26, N_{pooled} = 547).

Source data are available online for this figure.

© 2016 The Authors

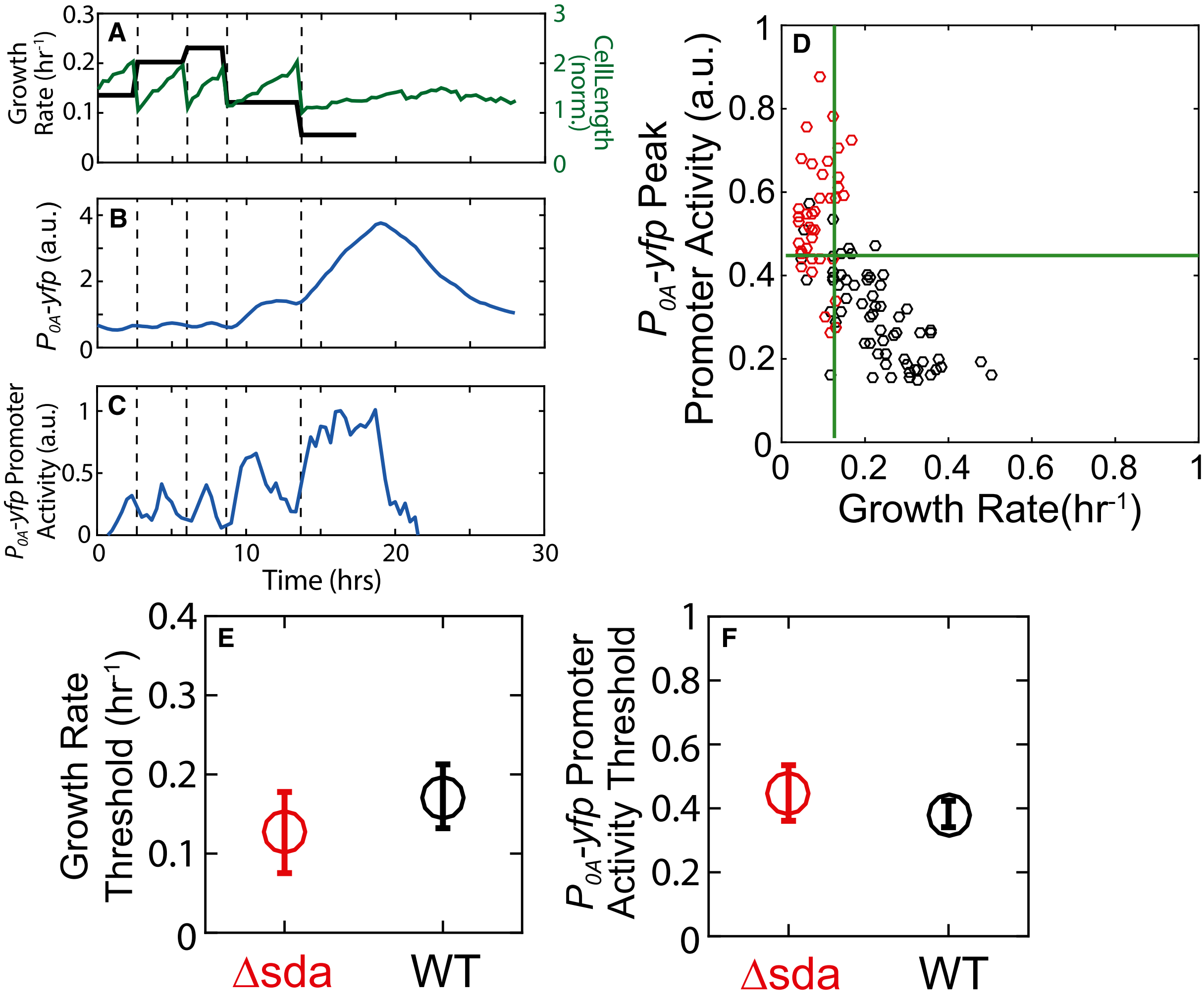

Figure EV6. Sda is not responsible for 0A activity pulsing or the growth threshold.

A–C Single-cell time-lapse microscopy using a P_{OA}-*yfp* reporter in a Δsda mutant. (A) Cell length (green) and its cell growth rate (gray), for a single cell over multiple cell cycles in starvation media. Similar to WT (see Fig 1D and E), in the Δsda mutant expression level of P_{OA}-*yfp* (B) increases in non-monotonic fashion and its promoter activity shows pulses with an increased amplitudes that is coordinated with a decrease in growth rate (C). In (A–C), vertical dashed lines indicate cell divisions.

D Measurements of P_{OA}-*yfp* promoter activity in the Δsda mutant show that 0A~P pulse amplitudes and growth rates are anti-correlated similar to WT. Each dot corresponds to measurements of the P_{OA}-*yfp* promoter activity pulse amplitude and growth rate of an individual cell cycle. Red and gray dots indicate cell cycles that end in sporulation and vegetative division, respectively. Green lines show the growth threshold (vertical line) and promoter activity threshold (horizontal line) determined using logistic regression similar to Fig 2D.

E, F Comparison of the WT versus Δsda growth thresholds (E) and promoter activity thresholds (F). Error bars show standard errors. Significance of strain dependence of sporulation thresholds was determined by logistic regression of pooled data (N_{pooled} = 449) and applying a *t*-test to determine whether the regression coefficient for the strain variable (= 0 for WT and 1 for Δsda) is zero (see Materials and Methods for details). Note that neither the growth threshold (*P*-value for strain coefficient = 0.17138, N_{pooled} = 449) nor the promoter activity threshold (*P*-value for strain coefficient = 0.41013, N_{pooled} = 449) depends on the strain.

Source data are available online for this figure.

 © 2016 The Authors
